# Generation of bispecific antibodies by structure-guided redesign of IgG constant regions

**DOI:** 10.3389/fimmu.2022.1063002

**Published:** 2023-01-10

**Authors:** Yordkhwan W. Iwasaki, Kannan Tharakaraman, Vidya Subramanian, Amnart Khongmanee, Andrew Hatas, Eduardo Fleischer, Troy T. Rurak, Patchara Ngok-ngam, Phanthakarn Tit-oon, Mathuros Ruchirawat, Jutamaad Satayavivad, Mayuree Fuangthong, Ram Sasisekharan

**Affiliations:** ^1^ Program in Environmental Toxicology, Chulabhorn Graduate Institute, Bangkok, Thailand; ^2^ Koch Institute for Integrative Cancer Research, Massachusetts Institute of Technology, Cambridge, MA, United States; ^3^ Translational Research Unit, Chulabhorn Research Institute, Bangkok, Thailand; ^4^ Center of Excellence on Environmental Health and Toxicology (EHT), Office of the Permanent Secretary (OPS), Ministry of Higher Education, Science, Research and Innovation (MHESI), Bangkok, Thailand; ^5^ Laboratory of Pharmacology, Chulabhorn Research Institute, Bangkok, Thailand; ^6^ Program in Applied Biological Sciences, Chulabhorn Graduate Institute, Bangkok, Thailand; ^7^ Department of Biological Engineering, Massachusetts Institute of Technology, Cambridge, MA, United States

**Keywords:** bispecific antibody (BsAb), Fc engineering, rational design, heterodimer, Fc receptors (FcR), HER2, EGFR – epidermal growth factor, structure-guided

## Abstract

Bispecific antibodies (BsAbs) form an exciting class of bio-therapeutics owing to their multispecificity. Although numerous formats have been developed, generation of hetero-tetrameric IgG1-like BsAbs having acceptable safety and pharmacokinetics profiles from a single cell culture system remains challenging due to the heterogeneous pairing between the four chains. Herein, we employed a structure-guided approach to engineer mutations in the constant domain interfaces (C_H_1-C_L_ and C_H_3-C_H_3) of heavy and κ light chains to prevent heavy-light mispairing in the antigen binding fragment (Fab) region and heavy-heavy homodimerization in the Fc region. Transient co-transfection of mammalian cells with heavy and light chains of pre-existing antibodies carrying the engineered constant domains generates BsAbs with percentage purity ranging from 78% to 85%. The engineered BsAbs demonstrate simultaneous binding of both antigens, while retaining the thermal stability, Fc-mediated effector properties and FcRn binding properties of the parental antibodies. Importantly, since the variable domains were not modified, the mutations may enable BsAb formation from antibodies belonging to different germline origins and isotypes. The rationally designed mutations reported in this work could serve as a starting point for generating optimized solutions required for large scale production.

## Introduction

Bispecific antibodies (BsAbs) are an attractive area of biologics as they combine the antigen binding fragments of two different monoclonal antibodies into one. In principle, they can be employed to 1) aggregate receptors on cell surface causing simultaneous inhibition of multiple signaling pathways (e.g., human epidermal growth factor receptor 2 (HER2) and HER3), 2) recruit immune cells to kill tumor cells (e.g. CD19×CD3), 3) minimize drug-resistance by binding to different epitopes on an antigen (e.g. viral protein), and 4) enhance antigen−specific targeting while avoiding off-target toxicities (1–6). In many scenarios, the effect of aggregating two or more targets within a spatiotemporal region often generates a synergistic effect (due to avidity) resulting in a superior therapeutic benefit than rendered by combination therapy involving the two parental monospecific antibodies (6).

Different BsAb formats exist, however, the vast majority of these formats suffer from one or more limitations: 1) involvement of a common light chain, which limits the number of antibodies that can be combined using this format, 2) alteration of the native IgG structure (e.g. single-chain variable fragment (ScFv)), which potentially affects the pharmacokinetic properties, 3) chemical conjugation of antibody fragments with potentially immunogenic linkers, 4) requirement of two master cell lines and additional time consuming and expensive *in vitro* techniques, 5) modification of the variable regions (V_H_/V_L_), which limits the utility to select antibody combinations (7–12). A hetero-tetrameric BsAb format that is broadly applicable (to different antibody combinations) and retains the intact structure, pharmacokinetics and bioavailability profiles of a regular monoclonal antibody is desired. In theory, a hetero-tetrameric BsAb can be generated by co-transfecting the heavy and light chains of two parental antibodies into a single cell culture system. However, this procedure generally results in a heterogeneous pairing of the four chains, resulting in potentially 10 different IgG products, out of which only one of them is the desired BsAb (**Supplementary Figure 1**) (13).

Multiple groups have developed technologies to aid cognate heavy-light pairing preference and Fc heterodimerization for proper bispecific assembly (**Supplementary Tables 1**, **2**). For example, Golay et al. created a tetravalent IgG-like bispecific using a C_H_1-C_L_ heterodimerization strategy (14). However, the format incorporates a peptidic linker to link the inner and outer Fab regions and thus deviates in size and geometry from conventional IgG antibodies. The heavy-light chain mispairing problem has been addressed through domain crossover (‘CrossMab’), where one Fab is untouched and the V_H_ or C_H_1 of the second Fab is switched with partner V_L_ or C_L_ domains. This technology is distinct from redesigning the Fab interface and has been successfully used to engineer multiple clinical candidates (15, 16). Other groups have employed camelid V_H_H as opposed to Fab or ScFv to bypass the heavy-light mispairing problem (17). Owing to the non-IgG like architecture, the drug metabolism and pharmacokinetic properties of these molecules may not resemble that of traditional IgG antibodies. Bönisch et al. elegantly designed C_H_1:C_L_ interface to prevent mispairing using the commercially available FoldX algorithm (18). The most commonly employed Fc heterodimerization technology makes use of the Knobs-into-Holes mutations (19). Since this, alternate solutions have been developed by many other groups (**Supplementary Table 1**). More recently, Leaver-Fay et al. employed computational docking and molecular modeling to develop heterodimeric Fc technology (20). Specifically, the heterodimerization was enabled by mutations that introduced hydrophobic/steric complementarity and electrostatic complementarity, much like Knob-into-Holes or electrostatic steering technology. However, like other studies in this category (**Supplementary Table 1**), it did not address the problem of heavy-light chain mispairing.

Herein, we employed a structure-guided approach to redesign the constant region interfaces C_H_1-C_L_ and C_H_3-C_H_3 of human IgG1κ antibodies to prevent mispairing of heavy-light chains and homodimerization of Fc heavy chains, thereby driving the kinetics to favor the formation of BsAb antibody. The mutations enabled the production of stable, full-length hetero-tetrameric BsAb in mammalian HEK cells with purity varying between 78%-85%, without compromising on the yield. The generated BsAbs showed dual-antigen binding and possessed intact Fc effector properties, such as binding to human Fc gamma receptor, C1q and FcRn. Despite the diverse solutions published to date, the positions/mutations identified by this study are unique, with only minimal overlap (e.g., K409R) (**Supplementary Tables 1**, **2** **)**, indicating the vastness of the solution space and potential room to innovate in this field. The C_H_1-C_L_ and C_H_3-C_H_3 interfaces identified by this study represent good starting points for further optimization (i.e., can be combined with other *de novo* or published mutations) to achieve purity levels required for production scale (>95%).

## Methods

### Capturing highly-networked protein residues (SIN)

The structural coordinates of C_H_1-C_L_ (PDB:3BKY) and C_H_3:C_H_3 (1L6X) were used to determine inter-chain inter-residue contacts including putative hydrogen bonds (including water-bridged ones), disulfide bonds, pi-bonds, polar interactions, salt bridges, and Van der Waals interactions (non-hydrogen) occurring between pairs of residues as described previously (21). These data were assembled into an array of eight atomic interaction matrices. A weighted sum of the eight atomic interaction matrices were computed to produce a single matrix that accounts for the strength of atomic interaction between residue pairs, using weights derived from relative atomic interaction energies. The inter-residue interaction network calculated in this fashion generates a matrix that describes all the contacts made between pairs of residues. Each element i, j is the sum of the path scores of all paths between residues i and j. The degree of networking score for each residue was computed by summing across the rows of the matrix, which corresponds to the extent of “networking” for each residue. The degree of networking score was normalized (SIN score) with the maximum score for each protein so that the score varies from 0 (absence of any network) to 1 (most networked). In alignment with our previous study, residues whose SIN score is above 0.4 is considered highly networked.

### Analysis of antibody interfaces and design of interfacial mutations

Bispecific interfaces (PDB: 1N8Z, 3P11 for C_H_1-C_L_, PDB: 1L6X for C_H_3-C_H_3) were analyzed with the help of 2D graph-based connectivity networks, a concept we had developed earlier (22). Although it was developed to analyze epitope-paratope interactions (22), the same methodology is applicable to other protein-protein interfaces. Basically, the inter-residue interactions between an interfacial residue (e.g., C_H_1) and its neighboring residues on its partnering domain (e.g., C_L_) are rendered in a 2D graph format as a visual aid, where nodes represent amino acids, and the edges represent inter-residue non-covalent interactions. Interactions considered include putative hydrogen bonds (including water-bridged ones), disulfide bonds, pi-bonds, polar interactions, salt bridges, and Van der Waals interactions (non-hydrogen) as described previously (21). Mutations or positions (when mutated) that contribute to more favorable or unfavorable interactions, as evaluated by this network analysis, were selected for experimental investigation.

### Site directed mutagenesis & DNA preparation

Site directed mutagenesis was performed using QuikChange II XL (Agilent Technologies). Sequence verified plasmid was amplified using PureLink HiPure Maxiprep Kit (Life Technologies).

### Cloning and design of BsAbs and mAbs

In this study, we designed two BsAbs, i.e., anti-EGFR/HER2 BsAb and anti-CD20/CD20 BsAb. The anti-EGFR/HER2 BsAb was constructed by combining a light and heavy chain of pertuzumab (PTAB), an anti-HER2 antibody, and a light and heavy chain of DL11, an anti-EGFR/HER3 antibody. The anti-CD20/CD20 BsAb was constructed by combining a light and heavy chain of rituximab (Rxm), a type I anti-CD20 antibody, and obinutuzumab, a type II anti-CD20 antibody. For improving the BsAb production, we mutated amino acids in C_H_1/C_L_ interface and C_H_3 constant regions. The DNA fragments of the desired heavy chains and light chains were generated by gene synthesis and cloned into pcDNA 3.3 expression vector. For the parental monoclonal antibodies, we cloned DNA coding pertuzumab or rituximab into pcDNA 3.1 (+) expression vector. The sequence of the inserted DNA fragment was confirmed by DNA sequencing.

### Antibody expression

We used the ExpiFectamine 293 transfection kit for transient transfection. First, HEK cells were seeded at 3.0 × 10^6^ cells/mL and incubated for 24 hours. After incubation, cells were harvested and diluted to 2.5 × 10^6^ cells/mL. For the preparation of transfection reaction, 80 µg of plasmid DNA was diluted in 4 mL of Opti-MEM medium (Gibco). In a separate tube, 219 µL of ExpiFectamine 213 reagent was diluted in 4 mL of Opti-MEM medium (Gibco). After the mixture was incubated for 5 minutes, the diluted DNA was added. Then, DNA-reagent complex was incubated for 20 minutes and added to HEK cell suspension. After incubation for 16-18 hours, 400 µL of Transfection Enhancers 1 and 4 mL of Transfection Enhancer 2, which were provided in transfection kit, were added. Cells were incubated for 6 days before harvesting.

### Protein purification

After transient transfection, antibodies in the cell culture supernatant were collected and filtered through the 0.2 µm Thermo Scientific Nalgene Rapid-Flow disposable filter unit with PES membrane (ThermoScientific). Antibodies were purified using HiTrap MabSelect Sure affinity column (GE Healthcare) using an AKTA Pure chromatography system (GE Healthcare). First, cell culture supernatant was loaded onto HiTrap MabSelect Sure affinity column pre-equilibrated with binding buffer (0.02 M phosphate, 0.15 M NaCl, pH 7.4). Protein was eluted using elution buffer (0.1 M sodium citrate, pH 3.0). Eluted fractions were collected in tubes containing 10% neutralization buffer (1 M Tris HCl, pH 8.8). For buffer exchange, protein sample was added into the filter column (30 kDa Ultra-4 Centrifugal Filter Units, Amicon) pre-washed with phosphate buffer saline, pH 7.4 (Gibco). Then, the filter column was centrifuged at 2800 g for 15 minutes at 4°C. This step was repeated until all of sample was loaded into filter column. Then, PBS was added into the filter column; and the filter column was centrifuged at 2800 g for 15 minutes at 4°C. This step was repeated until the elution buffer is less than 1%. After buffer exchange, the purified IgG was filtered using 0.2 µm filter in type II biological safety cabinet to obtain sterile antibodies for cell-based experiment.

### Sodium dodecyl sulfate polyacrylamide gel electrophoresis

The integrity of the antibody was also examined using reducing and non-reducing SDS-PAGE. For reducing condition, 600 ng of proteins in glycine/SDS solution (50 mM Tris-HCl pH 6.8, 2% SDS, 10% glycerol, 1% β-mercaptoethanol, 0.02% bromophenol blue) with 12.5 mM DTT were loaded on NuPAGE 4 to 12%, Bis-Tris, 1.0 mm, Mini Protein Gel, 12-well (ThermoFisher SCIENTIFIC). For non-reducing condition, 400 ng of proteins in glycine/SDS solution without DTT were loaded on NuPAGE 4 to 12%, Bis-Tris, 1.0 mm, Mini Protein Gel, 12-well (ThermoFisher SCIENTIFIC). Each sample was subjected to electrophoresis at 100 mA for approximately 120 min. Protein bands were visualized using InstantBlue^®^ Coomassie Protein stain (abcam) followed by destaining until the background was clear. The protein bands were visualized by ChemiDoc Touch Imaging System (BIO-RAD).

### Size exclusion chromatography

In this study, BsAbs and parental mAbs were subjected to size exclusion chromatography (SEC). Antibodies were diluted to the concentration of 5 mg/mL and filtered with 0.2 µM Nanosep MF Centrifugal Devices with Bio-Inert Membrane (PALL). SEC was performed using Agilent High Performance Liquid Chromatography (Agilent Technologies) consisting of a binary pump with a degasser, an autosampler was maintained at 4°C and a photo diode array detector. A TSKgel G3000SW_XL_ HPLC column (0.78 × 30 cm) (TOSOH CORPORATION) was operated at 25°C. The pre-filtered mobile phase was 0.2 M potassium phosphate buffer with 0.25 M potassium chloride at pH 6.2 with constantly at 0.7 mL/min. Antibodies were eluted isocratically over 11-14 minutes. The percentage of monomer and aggregates were calculated by area under the curve using OpenLab CDS 3.4 (Agilent). Mix standard proteins (ovalbumin; 43 kDa, p-aminobenzoic acid; 137.36 kDa ribonuclease A; 13.7 kDa, thyroglobulin; 660 kDa) were used for MW calculation of the antibodies.

### Mass spectrometry

Bispecific antibodies (BsAbs) were deglycosylated using Rapid PNGase F enzyme (NEW ENGLAND BioLabs) by incubating at 37°C for 18 hours prior intact mass analysis. Intact mass spectral data of deglycosylated BsAbs were acquired using an Orbitrap Q-Exactive HF Mass Spectrometer coupled to an Ultimate 3000 UHPLC system (Thermo Scientific). Buffer A is 0.1% formic acid in water and buffer B is 0.1% formic acid in acetonitrile. Approximately 3 µg of BsAbs were loaded to MAbPac RP Column (4 µm, 2.1 mm x 50 mm, Thermo Scientific) preconditioned with 20% buffer B. The first 2 minutes of gradient were maintained at 20% buffer B. The separation was attained using solvent gradient ramping from 20% to 65% buffer B over 4.5 minutes. Washing was done using 90% buffer B for 5 minutes. Column was then re-equilibrated with 20% buffer B for 5.5 minutes. The flow rate was set to 0.3 mL/min throughout all gradient steps. The column temperature was maintained at 80°C throughout all gradient steps. The HESI-II ion source parameters were set as following; spray voltage 4.20 kV, capillary temperature 325°C, S-lens RF 70%, sheath gas flow 50 units, and auxiliary gas flow rate 15 units. The data acquisition parameters were set as following; scan range m/z 800-4500, resolution 15,000, positive mode, FT Full scan AGC target 3×10^6^, fixed AGC mode, and maximum injection time 150 ms. BioPharma Finder version 3.0 (Thermo Scientific) software was used to deconvolute intact mass spectrum. An average molecular weight of BsAbs and their corresponding mispairing were calculated using Agilent MassHunter Sequence Manager B.09.00 (Agilent Technologies). Lysine clipping and glycine loss at C-terminus were considered as they are common variants found for antibody.

### Differential scanning calorimetry

The DSC measurements were obtained using Differential Scanning Calorimeter 3+ (DSC3+) Auto Sample Robot (Mettler Toledo). Antibodies were concentrated at 15 mg/mL in phosphate buffer pH 7.4. Antibodies were scanned from 30 to 95°C at a heating rate of 1°C/minute. The corresponding buffer was used as a baseline and subtracted from sample data. The thermal transition midpoint (T_m_) was obtained from the maximum temperature of the DSC thermogram. Data analysis was done using STARe Default DB V.16.

### Far-UV Circular dichroism spectroscopy

The Far-UV CD spectroscopy was performed to analyze secondary structure of antibodies using a Jasco J-815 spectropolarimeter (Jasco). The UV CD spectra were obtained in the intervals of 190-250 nm, in a quartz cuvette with a path length of 0.1 cm. The spectra were collected by continuous scanning at 0.1 nm intervals, a scanning speed of 50 nm/min, a response time of 1 s, a bandwidth of 1 nm, and 1 accumulation. The protein concentration was 0.1 mg/mL. The baseline was corrected in all experiments using a milliQ water as control.

### Sandwich ELISA

Dual-binding ELISA for EGFR and HER2 was developed to determine the binding activity of anti-EGFR/HER2 BsAb. A pre-absorbed Maxisorp 96-well plates (ThermoFisher SCIENTIFIC) was coated with 1 μg/mL of human EGFR protein (Sino Biological) in phosphate-buffered saline (PBS) and incubated at 4°C overnight. Plate was washed three times with PBS. A blocking solution of 1% BSA in PBS plus 0.05% tween (PBST) was added. Then, the plate was incubated at room temperature for 1 hour and washed three times by PBST. Serial dilution (2.5-fold) of the anti-EGFR/HER2 BsAb was done with the starting concentration of 8 µg/mL for 11 dilutions. BsAb was diluted in PBS and in duplicate with 3 different batches of production. The plate was incubated at room temperature for 2 hours and then washed with PBST. Incubated plate was then wash three times with PBST and added with 100 µL of 1 µg/mL human HER2 protein (Sino Biological) to each well. Plate was incubated at room temperature for 2 hours and washed three times with PBST. After that, 0.1 µg/mL of secondary antibody (rabbit monoclonal anti-human HER2 antibody (HRP) (Sino Biological) was added. Plate was incubated at room temperature for 1 hour and then washed with PBST. For detection, TMB substrate (SeraCare) was added and incubated for 5 minutes. The reaction between TMB substrate and HRP on secondary antibody was stopped by adding 1N sulfuric acid. Plate was read using a microplate reader (SpectraMax iD3) at 450 nm.

### HER2 binding ELISA

HER2 in phosphate-buffered saline at concentration of 1 μg/mL was immobilized on Maxisorp 96-well plate (ThermoFisher SCIENTIFIC) at 4°C overnight. The antigen coated plate was then washed with PBS three times to remove excess antigens. Plate then was blocked with 1% BSA in PBST for 1 hour following by incubation with serially diluted antibodies with the starting concentration of 8 µg/mL (2.5-fold) for 2 hours. A total of 100 µL/well of goat anti-human IgG H&L (abcam; 1/12500 dilution) was added to plate and incubated for 1 hour. Washing 3 times with PBST was performed after each incubation step. After final wash, the plates were developed with TMB (3,3’,5,5’-tetramethylbenzidine) substrate (SeraCare). After the reaction between TMB and HRP was stopped by 1N sulfuric acid, absorbance at 450 nm was recorded using a microplate reader (SpectraMax iD3).

### Surface plasmon resonance binding assay

SPR studies were performed on a Biacore T200 instrument (GE Healthcare). Each Fc receptor was captured on a pre-immobilized with His capture kit (Cytiva) Biacore chip for kinetic studies of bispecific antibodies. Receptors used in this study were FcɣRIIIa V and F forms, FcRn, and C1q. For FcɣRIIIa binding, either 0.2 µg/mL human FcɣRIIIa V form (Human CD16a/FcɣRIIIa Protein (176 Val, His Tag), Sino Biological) or F form (CD16a Protein, Human, Recombinant (176 Phe, His Tag), Sino Biological) in Biacore buffer HBS-EP+ (10 mM HEPES, pH 7.4, 150 mM NaCl, 3 mM EDTA, 0.05% Surfactant P20, Cytiva) was run through a pre-His capture immobilized series S sensor chip CM5 (Cytiva). Later, five concentrations of each BsAb in Biacore buffer HBS-EP+ was flowed through the FcɣRIIIa-immobilized CM5 sensor chip. The binding was measured in association and dissociation phases at 30 µL/min.

For FcRn binding, Human FcRn (Recombinant Human FcγR/FcRn protein, Sino Biological) was diluted to 0.25 µg/mL in Biacore buffer HBS-EP+ (Cytiva) and was run through a pre-captured series S sensor chip CM5 (Cytiva). Serial dilution (2-fold) of BsAbs were done with the starting concentration of 2000 nM for 5 dilutions in Biacore buffer HBS-EP+ (10 mM HEPES, pH 6, 150 mM NaCl, 3 mM EDTA, 0.005% Surfactant P20, Cytiva) and run kinetic binding through previously FcRn-immobilized sensor chip. The binding was measured in association and dissociation phases at 30 µL/min. For C1q binding of anti-CD20/CD20 BsAb, human C1q (Native Human C1q protein, abcam) was diluted to 120 µg/mL in immobilization buffer (Cytiva) by using amine coupling kit (Cytiva) and was immobilized on a series S sensor chip CM5 (Cytiva). Serial dilution (2-fold) of anti-CD20/CD20 BsAb was done with the starting concentration of 8000 nM for 5 dilutions in Biacore buffer HBS-EP+ (10 mM HEPES, pH 7.4, 150 mM NaCl, 3 mM EDTA, 0.005% Surfactant P20, Cytiva) and run kinetic binding through previously C1q-immobilized sensor chip. The binding was measured in association and dissociation phases at 30 µL/min.

The C1q binding of anti-EGFR/HER2 BsAb was done by a sandwich Biacore assay. The anti-EGFR/HER2 BsAb was diluted in Biacore buffer HBS-EP+ pH 7.4 (Cytiva) to a concentration of 1 µg/mL and was captured on a sensor chip Protein L (Cytiva). Serial dilution (2-fold) of human C1q protein was done with the starting concentration of 5 nM for 5 dilutions in Biacore buffer HBS-EP pH 7.4 (Cytiva) and was flowed over the previously captured chip at 30 µL/min. All sensogram data were processed and analyzed by Biacore Evaluation software (GE Healthcare).

## Results

### Structure-guided redesign of IgG1κ constant domains

We employed the structures of IgG1κ anti-HER2 (PDB: 1N8Z) and anti-HER1/3 (PDB: 3P11) antibodies to design and screen for Fab (i.e., C_H_1-C_L_) and Fc (C_H_3-C_H_3) mutations. These two antibodies were chosen since their heavy and light chains possessed an intrinsic ability for heterogeneous pairing, thus presenting an excellent test case to screen for mutations that enable selective chain pairing preferences. Since the predicted Fab and Fc mutations were studied separately, the screening process for Fab mutations made use of WT Fc, and similarly, the screening process for Fc mutations employed WT Fab.

### Computational design and screening of C_H_1-C_L_ mutations to prevent mispairing between heavy and light chains

The C_H_1-C_L_ and V_H_-V_L_ domain pairs are each related by a pseudo-2-fold symmetry. Therefore, we attempted to engineer Fab interface mutations that would introduce asymmetry in the mispaired heavy-light chain interfaces, while retaining WT-like symmetry in the cognate heavy-light chain interfaces. With this design approach, it is expected that the heavy (or light chain) of the first antibody would pair with its cognate chain with higher affinity and specificity than to the non-cognate chain of the second antibody. To aid in the design process, we chose to mutate C_H_1-C_L_ interface residues (**Supplementary Table 3**) that were 1) highly interconnected by a network of non-covalent interactions or have high SIN scores (Methods), 2) proximal to the pseudo-2-fold axis (**Supplementary Table 4**), and 3) evolutionarily conserved across Ig genes. Criteria 1 & 2 ensure that the mutations have a profound impact on the interface thus minimizing the need to make mutations in the variable domains. Criteria 3 ensures that the mutations are not specific to the system under consideration but are transferrable to other antibody combinations and isotypes. Using computational modeling, we explored the possibility of engineering two sets of oppositely charged residues, one on each Fab, such that it would create a repulsive charge-charge interaction (asymmetry) in the context of heavy-light chain mispairing. For example, the interacting residue pair Leu150 (C_H_1) – Val132 (C_L_) in the WT interface would be substituted Asp150 (C_H_1′) – Lys132 (C_L_′) in the first Fab and Arg150 (C_H_1′′) – Asp132 (C_L_′′) in the second Fab. In this scenario, the interface between mispaired heavy-light chains will be destabilized as a result of bringing two like charges into close proximity. Likewise, we also explored the possibility of introducing two sets of “knobs” and “holes” in place of a C_H_1-C_L_ residue pair, one on each Fab, which would lead to a sterically unfavorable interaction (asymmetry) in the context of heavy-light mispairing. Then, we combined two or more C_H_1-C_L_ mutation sets, where structurally feasible, to generate additional sets of C_H_1-C_L_ mutations. A total of 44 C_H_1-C_L_ mutations (referred henceforth as “clusters”) were generated with the help of the structure-guided process (Methods) (**Supplementary Table 5**).

**Figure 1:**
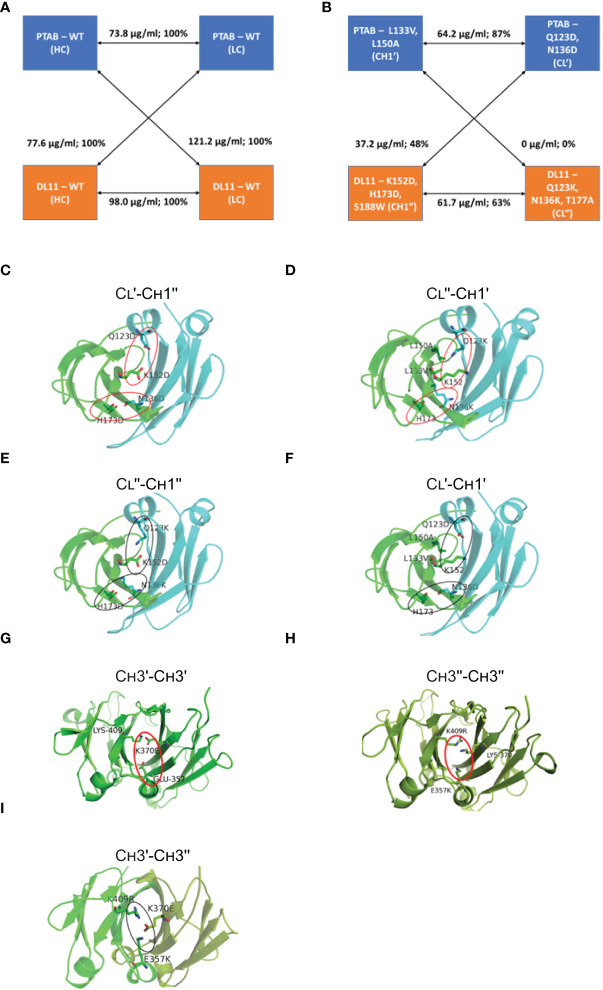
Structure-guided redesign of C_H_1-C_L_ interface for BsAb engineering. Expression levels of cognate paired and mispaired monospecific species formed by heavy and light chains of pertuzumab (PTAB) and DL11 **(A)** without mutations and **(B)** with mutations. Rendered in **(C-F)** are the 3D derived models of the C_H_1 and C_L_ domain interface observed in PDB: 3BKY, where the view is perpendicular to the pseudo-2-fold axis. The C_H_1 and C_L_ domains are colored in green and cyan, respectively. The side chains of the modified C_H_1-C_L_ interface residues and WT residues that aid or perturb the C_H_1-C_L_ interface are represented as sticks and labeled. Attractive and repulsive interactions are circled using black and red ovals, respectively. **(G–I)** Network and two-fold axis relationship of mutants to enhance heterodimerization of C_H_3 domains in BsAbs. The view is looking down on the 2-fold axis of symmetry. The C_H_3 domains of the two antibodies are colored in green and splitpea, respectively. Potential repulsive interactions that emerge in C_H_3 homodimers, such as Glu357- Lys370Glu **(G)** and Glu357Lys - Lys370, Lys409Arg - Lys370 (**H**), are highlighted by red oval, whereas the interactions that enhance the heterodimer formation in the BsAb are highlighted by black oval (**I**). Note that the instance of the interactions that is away from the reader is not shown, for clarity.

Next, we assessed the potential of a predicted C_H_1-C_L_ cluster to promote preferential pairing between cognate chains by expressing the two correctly paired and two mispaired monospecific antibody species (**Supplementary Figure 2**) and then quantifying their expression using IgG ELISA (**Supplementary Table 5**). **Supplementary Table 5** captures the expression levels of matched and mismatched antibodies relative to their unmodified versions. From this, the cluster that led to maximum expression difference between the correctly paired and mispaired species was identified (**Supplementary Table 5**). In many cases, mutations that lowered the expression levels of the mispaired IgG species also caused moderate-to-significant impact on the expression of the correctly paired IgG species (e.g., clusters 1, 17, 18, 43 in **Supplementary Table 5**). In other cases, mutations that maintained the expression levels of the correctly paired IgG species were not detrimental to the mispaired species (e.g., clusters 23, 30, 35 in **Supplementary Table 5**). Cluster 40 yielded by far the most favorable results (in **Supplementary Table 5** and **Table 1** **).** In this context, the correctly paired-IgG species expressed at 87% (C_L_′-C_H_1′) & 63% (C_L_′′-C_H_1′′) of their unmodified versions, whereas the mispaired-IgGs expressed at much lower levels of 0% (C_L_′′-C_H_1′) and 48% (C_L_′-C_H_1′′) (**Figures 1A, B** **)**. Critically, one of the mispaired species represented by C_L_′′ and C_H_1′ chains expressed remarkably poorly, indicating the potential of this cluster to reduce or eliminate the formation of n=3 mispaired species from the mixture of ten (**Supplementary Figure 1**). This preferential pairing is likely due to the (1) repulsive interactions created by two like-charged residues– Lys152Asp (C_H_1′′)-Gln123Asp (C_L_′), His173Asp (C_H_1′′)-Asn136Asp (C_L_′) (**Figure 1C**). Likewise, structural modeling indicates that the interface between C_H_1′- C_L_′′ will be destabilized by two unfavorable interactions involving like-charged residues (1) Lys152 (C_H_1′) and Gln123Lys (C_L_′′) and (2) His173 (C_H_1′) and Asn136Lys (C_L_′′) (**Figure 1D**). In the context of the cognate heavy-light interface (C_L_′′- C_H_1′′)κ, Lys152Asp and His173Asp of C_H_1′′ will form favorable interactions with the oppositely charged Gln123Lys and Asn136Lys of C_L_′′, respectively (**Figure 1E**). Likewise, the interface formed by C_L_′ and C_H_1′ will be stabilized by the favorable electrostatic interactions between (1) Lys152 (C_H_1′) and Gln123Asp (C_L_′) and (2) His173 (C_H_1′) and Asn136Asp (C_L_′). The Leu133Val and Leu150Ala mutations on C_H_1′ create a void to accommodate the necessary Gln123Asp mutation on C_L_′ (**Figure 1F**).

**Table 1:** C_H_1-C_L_ mutations that favor the formation of cognate heavy-light pairing over mispairing. The C_H_1-C_L_ of pertuzumab and DL11 are referred as C_H_1′-C_L_′ and C_H_1′′-C_L_′′, respectively.

**Pertuzumab**	Q123D, N136D (C_L_′)	L133V, L150A (C_H_1′)
**DL11**	Q123K, N136K, T177A (C_L_′′)	K152D, H173D, S188W (C_H_1′′)

Next, we evaluated the specificity afforded by the C_H_1-C_L_ mutations using a 3-chain competition assay where two heavy chains (cognate and non-cognate) were forced to compete with a light chain for assembly (Methods). The pairing preference between the chains was evaluated using LC-MS. In the absence of the C_H_1-C_L_ mutations, 3 species are expected to form, a WT antibody, a mispaired monospecific and a common light chain BsAb. Along similar lines, competition experiments were conducted using two light chains and a heavy chain, where three species are expected to form in the absence of C_H_1-C_L_ mutations (WT antibody, mispaired monospecific and a common heavy chain BsAb). Mutations L133V, L150A (C_H_1′) and Q123K, N136K, T177A (C_L_′′) completely prevented heavy-light mispairing, suggesting its potential to reduce the number of mispaired species in the context of the actual hetero-tetrameric BsAb assembly (**Supplementary Table 6**).

### Computational design and screening of C_H_3-C_H_3 mutations to prevent homodimerization of Fc heavy chains

Several solutions have been proposed to prevent the homodimerization of Fcs. The most prominent is the knob-into-holes mutations (19), which generates antibody heavy chains for heterodimerization, using steric complementarity.

Our design strategy centers around the four ionic interactions in the C_H_3-C_H_3 interface (Asp356–Lys439, Glu357–Lys370, Lys392-Asp399 and Asp399–Lys409) (23). Each ionic interaction pair occurs twice in the C_H_3-C_H_3 interface as a result of the two-fold symmetry. The designs aim to disrupt the ionic interaction pair within the homodimers through a hydrophobic substitution. For instance, Asp356–Lys439 was made Asp356–Phe439 in the first antibody and Val356–Lys439 in the second antibody. The switch in the side chain character (ionic to hydrophobic) is expected to reduce the propensity for homodimerization, while favoring heterodimerization. The newly formed Fc heterodimer will contain one ionic and one hydrophobic pair instead of two ionic interactions as seen in a homodimer. Similarly, we made attempts to create asymmetry by introducing charged residues instead of hydrophobic. Where possible, designs from the two strategies were combined together in order to generate profound effects on Fc pairing.

**Figure 2:**
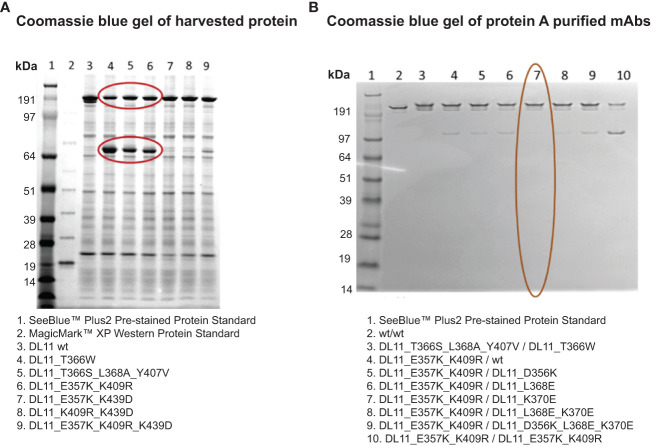
Identification of lead C_H_3 mutations for Fc heterodimer formation using non-reducing SDS-PAGE analysis. Non-reducing SDS-PAGE of representative Fc engineered antibodies are shown in **(A)**. Protein samples were harvested. Gel was stained with Coomassie blue. Mutations that induce the formation of half-antibody fragments are circled. These include the published knobs-into-holes mutations **(**lanes 4 & 5**)** and the computationally engineered variant (lane 6).**(B)** Identification of compensatory Fc mutations using a three-chain system (2 heavy and 1 light). The Fc mutations shown in column 7 (circled in red) show maximum reduction in the formation of half-antibody fragments, similar to the traditional knob-into-holes mutations (column 3). Lane 1 in **A** and **B** contains SeeBlue protein standards.

Our criteria for screening the Fc mutations was based on the observation that transient expression of a heavy and light chain carrying the traditional Fc knob mutation (19) generates half-antibody **(**H-L**)** fragments along with the full IgG **(** **Figure 2A**). Using the same principle, we determined potential knob-like mutation**(**s**)** from the list of predicted mutations based on the ability to induce the formation of half-antibody fragments. Coomassie gel showed the Fc mutations Glu357Lys, Lys409Arg formed high amounts of half-antibody fragments, suggesting that these mutations possess knob-like features **(** **Figure 2A**). Keeping the knob mutation fixed, we then evaluated potential compensatory mutations **(**hole-like**)** based on their ability to reduce the formation of half-antibody fragments (*via* heterodimerization) in a competition 3-chain system **(**2 heavy, 1 light**).** Our analysis showed that maximum reduction in half-antibody fragments occurred in the presence of compensatory mutation, Lys370Glu (**Figure 2B**). Structural modeling of these mutations on the C_H_3-C_H_3 interface provides a rationale for the preferential heterodimer formation. The formation of homodimers (C_H_3′-C_H_3′; C_H_3′′-C_H_3′′) is prevented by the repulsive charges that are brought close together (**Figures 1G, H**) whereas the heterodimer formation (C_H_3′-C_H_3′′) is aided by favorable ionic interactions between closely-packed residues (**Figure 1I**). Hence, these two sets of mutations were employed for preventing homodimerization (**Figures 1G–I**).

Our structure-guided design process introduces mutations in the conserved regions of an IgG antibody. In order to assess the potential impact of the engineered mutations on increasing immunogenicity risk, we employed NetMHCIIpan version 4.0 (24) to predict MHC II binding sites on the WT and modified constant region sequences for common HLA class II alleles: DRB1_0101, DRB1_0701, DRB1_0901, DRB1_1101, DRB1_1201, DRB1_1501, DRB1_0401, DRB1_0405, DRB1_0301, DRB1_1302, DRB3_0101, DRB3_0202, DRB4_0101, and DRB5_0101 (25). The software was run using default settings. Peptides of length n=15 were searched. The software compares the predicted affinity of a given peptide with the scores of 200,000 random natural peptides of the same length and computes a percentile rank. Then, it classifies the peptide as a weak binder or a strong binder if the percentile rank is below 5% or 1%, respectively. **Supplementary Table 7** lists the number of weak and strong binders in the WT and modified constant regions. Remarkably, the designs led to a net decrease in the number of weak and strong binders (**Supplementary Table 7**). Notably, mutations in 3/4 chains led to a decrease in MHC II binders. On the other hand, Q123K, N136K, and T177A in the light chain led to a modest increase in the number of MHC II binders; additional studies with therapeutic candidate molecules will be needed to confirm whether these mutations will have a detrimental impact (e.g., anti-drug antibody response).

To determine whether these mutations will have any impact on solubility, we employed CamSol (26) to predict the solubility of WT C_H_1-C_L_ and C_H_3-C_H_3 regions and their mutants. CamSol has been successfully employed for rational design of protein variants having improved solubility (26). The goal of this exercise was to determine whether the introduced changes will lead to a decrease in solubility character. CamSol was run using the homology models of different constant region interfaces (C_H_1-C_L_ (WT), C_H_1′-C_L_′, C_H_1′′-C_L_′′, C_H_3-C_H_3 (WT), C_H_3′-C_H_3′′) under default parameter settings: pH 7 and patch radius 10. Interestingly, the structurally-corrected intrinsic solubility scores of the modified interfaces were higher than that of the native interface, suggesting that the introduced mutations will not lead to reduced solubility (or increased hydrophobicity). Specifically, the solubility scores for C_H_1-C_L_ (WT), C_H_1′-C_L_′, C_H_1′′-C_L_′′, C_H_3-C_H_3 (WT), and C_H_3′-C_H_3′′ were found to be -0.055342, 0.103082, 0.114590, 0.572991, and 0.609848, respectively.

### Characterization of BsAbs

We employed anti-EGFR/HER2 and anti-CD20/CD20 as two systems to study and understand the utility of the mutations (**Supplementary Figure 3**). For the anti-EGFR/HER2 BsAb, we employed the variable domain sequences of pertuzumab (anti-HER2) (27) and duligotuzumab/DL11 (anti-EGFR/HER3) (28). Previous studies have shown that simultaneous targeting of EGFR, HER2, and HER3 with multispecific antibodies can prevent compensatory tumor promoting mechanisms within the HER family, providing a scientific rationale for the development of a pan-HER antibody (29). For the anti-CD20/CD20 BsAb, we used the variable domain amino acid sequences from the type I anti-CD20 monoclonal antibody, rituximab (30), and a type II anti-CD20 monoclonal antibody, obinutuzumab (31).

**Figure 3:**
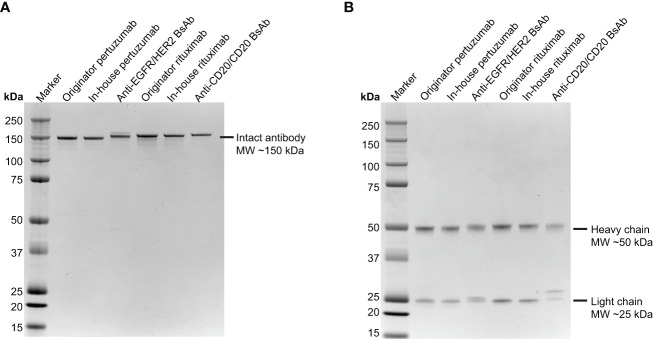
SDS-PAGE analysis of the two BsAbs compared to parental mAbs. Purified pertuzumab, anti-EGFR/HER2 BsAb, rituximab and anti-CD20/CD20 BsAb were subjected to electrophoresis in 4-12% SDS PAGE under **(A)** nonreducing condition and **(B)** reducing condition then stained with Coomassie Blue. Precision Plus Protein Kaleidoscope Prestained Protein Standards (Bio-Rad) was used as molecular weight marker.

**Figure 4:**
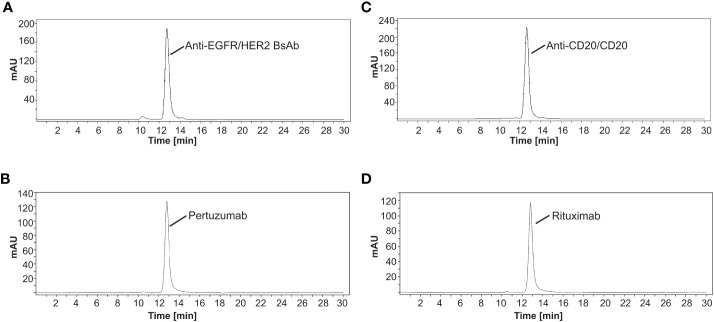
Separation of antibodies by size exclusion chromatography. The elution profiles showed a major peak of **(A)** anti-EGFR/HER2 BsAb, **(B)** pertuzumab, **(C)** anti-CD20/CD20 BsAb, and **(D)** rituximab with a retention time that is consistent with their expected molecular weight of around 144-145 kDa, respectively. All SEC experiments were done in triplicate. Chromatograms shown here are representative of the results.

Briefly, the EGFR/HER2 and CD20/CD20 BsAbs were expressed in ExpiFectamine 293 transfection system and purified using protein A column chromatography as described in Methods. **Supplementary Table 8** contains the amino acid sequences of the two bispecific antibodies that were expressed. After purification, protein integrity was determined by SDS-PAGE together with Coomassie blue staining. Under non-reduced conditions, anti-EGFR/HER2 and anti-CD20/CD20 BsAbs showed a major band at around 150 kDa, which was similar to the parental mAbs, (**Figure 3A**). Under reducing conditions, both BsAbs bands around 50 kDa and 25 kDa corresponding to the heavy and light chains, respectively. As expected, the parental mAbs also showed two bands corresponding to the heavy and light chains (**Figure 3B**). Analytical size exclusion chromatography (SEC) was performed to assess the homogeneity of the bispecific antibodies as well as parental mAbs. Both BsAbs were predominantly (>95%) monomeric in content. All antibodies eluted from the column with retention times that are consistent with their expected molecular weight of around 144-145 kDa (**Figure 4**).

**Figure 5:**
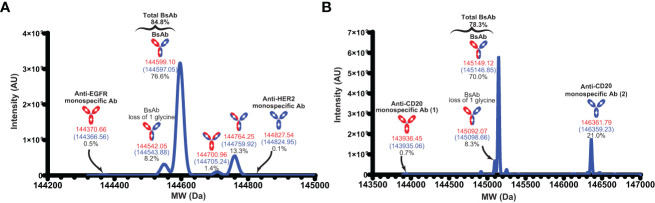
Intact mass analyses. **(A)** The deconvoluted MW of the anti-EGFR/HER2 BsAb. **(B)** The deconvoluted MW of the anti-CD20/CD20 BsAb. MWs in red indicate the theoretical mass with C-terminal lysine clipping and MWs in blue indicate the observed mass. Anti-CD20 monospecific Ab (1) and anti-CD20 monospecific Ab (2) contain Fab corresponding to rituximab and obinutuzumab monoclonal antibodies, respectively.

To further characterize the BsAbs, intact mass analyses were performed using mass spectrometry. In case of the anti-EGFR/HER2 BsAb, the observed mass of this main peak (144597.05 Da) (**Figure 5A**) was close to the theoretical calculated mass for the anti-EGFR/HER2 BsAb with C-terminal lysine clipping (144599.10 Da) with 14.18 ppm mass difference. We also observed a peak corresponding to a variant (glycine loss) of the BsAb. Taken together, the purity of the correct forms of anti-EGFR/HER2 BsAb was 84.8% (**Figure 5A**). In addition to these two peaks, we observed four distinct peaks corresponding to other species: two monospecific antibodies (0.6%), single (1.4%) and dual (13.3%) pertuzumab light chain mispairs (**Figure 5A**). In the anti-CD20/CD20 BsAb example, the mass of the major peak was 145146.85 Da with 15.64 ppm difference from the theoretical calculated mass of the BsAb with C-terminal lysine clipping (145149.12 Da) (**Figure 5B**). Like in the case of the anti-EGFR/Her2 BsAb, we observed a neighboring peak corresponding to a glycine loss variant of anti-CD20/CD20 BsAb. Together, the purity of the correct forms of anti-CD20/CD20 BsAb was 78.3% (**Figure 5B**). Besides these, we also observed peaks corresponding to the two parental monospecific antibodies which accounted for a total of 21.7% of the sample (**Figure 5B**). While we observed pertuzumab light chain mispairs (but not DL11 light chain mispairs) in the case of anti-EGFR/Her2 BsAb, the profile for the anti-CD20/CD20 BsAb shows complete absence of light chain mispairs. This observation also indicates that the chances of formation of dual light chain mispaired species, which has the same mass as the BsAb, are unlikely in both cases. The byproducts can be controlled or minimized through additional Fab/Fc interface engineering, alteration of chain transfection ratios (20), introduction of protein A ablation mutation (32), pI engineering (33), and/or other secondary purification techniques.

**Figure 6:**
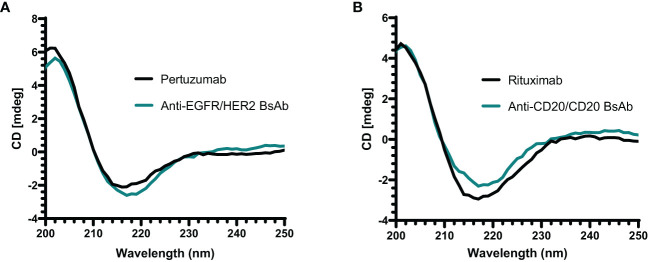
Far-UV CD spectrum of antibodies. **(A)** The spectrum of anti-EGFR/HER2 BsAb and its parental mAb (pertuzumab) show a negative band at around 218 nm. **(B)** The anti-CD20/CD20 BsAb and its parental mAb (rituximab) also show a similar spectrum of negative band as anti-EGFR/HER2 BsAb at approximately 218 nm. All proteins were buffer exchanged in milli-Q water prior analysis and done in triplicate.

The secondary structure of anti-EGFR/HER2 and anti-CD20/CD20 bispecific antibodies was assessed by far-UV CD analysis (200 to 240 nm). The spectrum of both BsAbs showed a similar negative maximum at approximately 218 nm similar to their parental mAbs (**Figure 6**). These results demonstrate that anti-EGFR/HER2 and anti-CD20/CD20 bispecific antibodies predominantly contain beta-sheet consistent with the quaternary structure of an IgG molecule (34–36).

**Figure 7:**
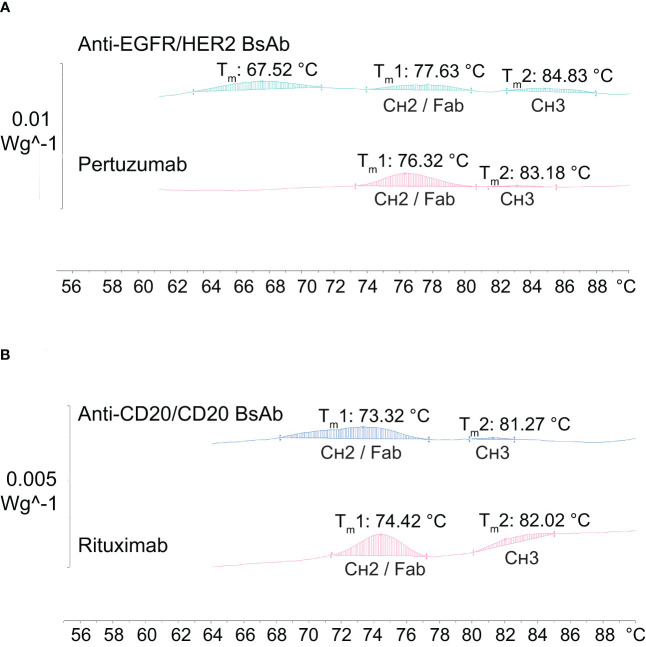
Thermal stability analysis of antibodies by differential scanning calorimetry (DSC). **(A)** The anti-EGFR/HER2 BsAb together with its parental mAb (pertuzumab) show two peaks of similar T_m_ values corresponding to the C_H_2/Fab and C_H_3 domains, with an extra peak at T_m_ 67.52°C. **(B)** The anti-CD20/CD20 BsAb exhibits similar T_m_ values for the C_H_2/Fab and C_H_3 domains when compared to its parental mAb (rituximab).

Next, the thermal stability profile of the two purified BsAbs was assessed by using differential scanning calorimetry (DSC) and compared to their parental mAbs. A mid-point temperature (T_m_) of BsAbs, which represents the temperature at which 50% of the protein is in its native conformation and 50% in denatured conformation, was used as an indicator for thermal stability. As shown in **Figure 7A**, the T_m_ values of the anti-EGFR/HER2 BsAb were observed to be 77.63°C for the C_H_2/Fab domains and 84.83°C for the C_H_3 domain, which were similar to the parental monospecific antibody pertuzumab, which has the wild-type sequence (C_H_2/Fab: 76.32°C; C_H_3: 83.18°C). Another (third) peak with T_m_ at 67.52°C was found in the anti-EGFR/HER2 (**Figure 7A**). This is likely to correspond to the Fab/C_H_2 region of the second parental monospecific antibody DL11 and/or the contaminants carrying the heavy chain of DL11 and light chain of pertuzumab (**Figure 5A**). This is supported by the fact that DL11 is reported to have lower T_m_ than pertuzumab (37). For the anti-CD20/CD20 BsAb, the T_m_ values were observed at 73.32°C for C_H_2/Fab domains and 81.27°C for C_H_3 domain, which were similar to the parental monospecific antibody rituximab, which has the wild-type sequence (C_H_2/Fab: 74.42°C; C_H_3: 82.02°C) (**Figure 7B**). Taken together, the T_m_ values of the two BsAbs are similar to their parental mAbs, as well as, in the range observed for naturally occurring antibodies, indicating that the mutations do not lead to significant destabilization (38, 39).

### Fab binding activity of anti-EGFR/HER2 BsAb

**Figure 8:**
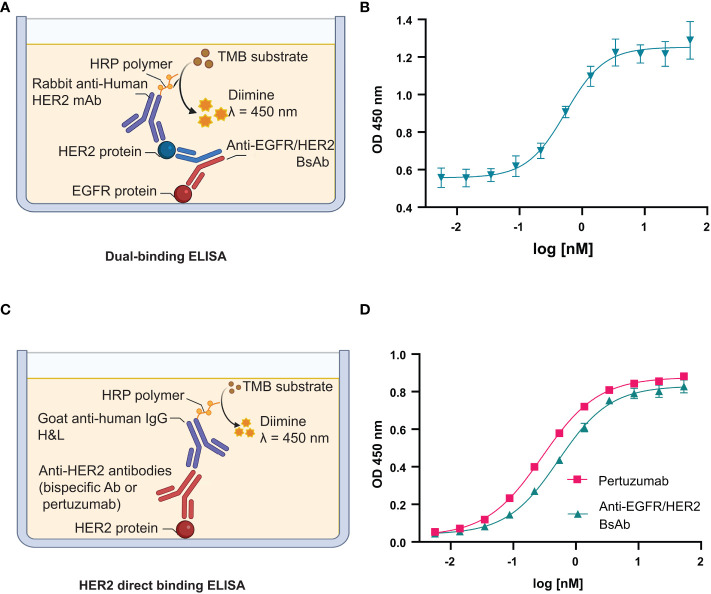
Fab binding activity of anti-EGFR/HER2 BsAb. **(A)** Schema of dual-binding ELISA format that was used in this study. **(B)** The anti-EGFR/HER2 BsAb shows dose-dependent binding consistent with its proposed quaternary structure. **(C)** Schema of direct HER2 binding ELISA. **(D)** The binding affinity of BsAb was compared to pertuzumab.The error bars indicate standard deviation from three independent experiments. Graphs were generated using GraphPad Prism version 8.4.3 for Windows.

We evaluated the dual antigen binding capabilities of the BsAb using a sandwich ELISA, where the detection signal from the secondary antibody is obtained only when the BsAb is bound to both target antigens (**Figures 8A, B**) (Methods). As shown in **Figure 8B**, the anti-EGFR/HER2 BsAb bound to their target proteins in a dose-response manner. These results indicate that the modifications made to the constant regions did not impact the Fab-based binding activity. The anti-CD20/CD20 (type ½) was not amenable to a sandwich ELISA. We also performed the HER2-binding ELISA to confirm that anti-EGFR/HER2 BsAb can bind to HER2 antigen **Figures 8C, D**. The EC_50_ values of BsAb and pertuzumab were 0.41 and 0.26 nM, respectively (**Figure 8D**). The HER2 binding EC_50_ values of the bispecific antibody was 1.6 times weaker than that of pertuzumab, indicating no major loss in binding as a result of the new format.

### Fc binding activity of BsAbs

**Figure 9:**
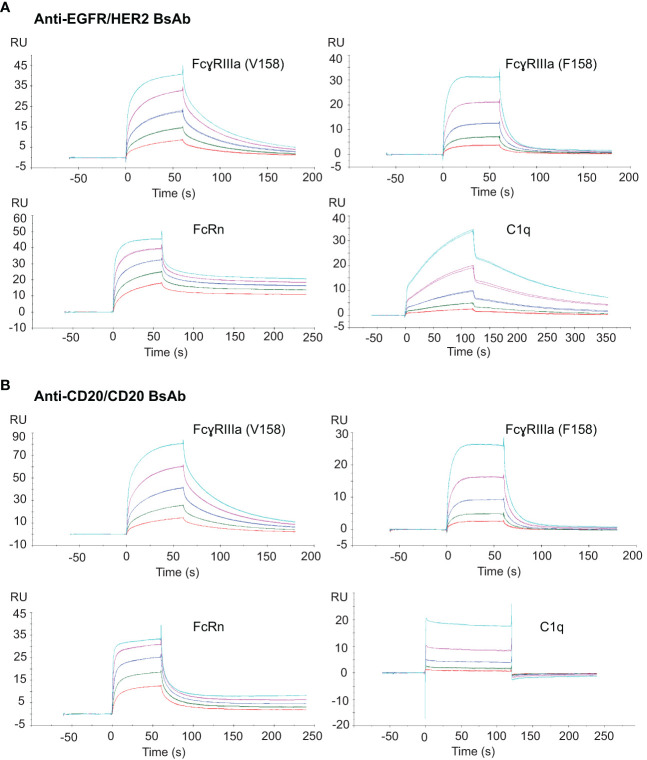
Fc binding activity of the two BsAbs to their target proteins. **(A)** SPR sensograms of soluble anti-EGFR/HER2 BsAb to chip-capture FcɣRIIIa (V158), FcɣRIIIa (F158), FcRn and the binding of this BsAb coated on protein-L chip to a soluble C1q protein. **(B)** SPR sensograms of soluble anti-CD20 BsAb to chip-capture FcɣRIIIa (V158), FcɣRIIIa (F158), FcRn and C1q protein. All Fc receptor SPR bindings were done in triplicate.

Alterations to the Fc peptide backbone or glycan structure are known to critically affect the effector properties (CDC and ADCC) and FcRn-dependent pharmacokinetics of IgG1 therapeutic antibodies (40). To determine whether the introduced mutations had any impact on binding to the effector molecules, surface plasmon resonance (SPR) was used to measure the binding activity of the two BsAbs to Fc receptors including FcɣRIIIa V and F forms, FcRn, and C1q. The binding of anti-EGFR/HER2 BsAb is shown in **Figure 9A**. The equilibrium dissociation constants (*K*_D_) for binding to immobilized human FcɣRIIIa V form (V158) and F form (F158) were 185 ± 5.3 nM and 735.8 ± 27.4 nM, respectively. The *K*_D_ of FcRn binding to anti-EGFR/HER2 BsAb was 11.5 ± 2.5 nM. To measure complement binding, soluble human C1q was run though the BsAb-captured protein-L chip. The *K*_D_ was measured at 6.4 ± 0.3 nM. Binding of soluble Fc receptors and C1q to human IgG1 monospecific antibodies has been extensively studied (41–45). The equilibrium dissociation constants (*K*_D_) we determined match closely with literature (41–45), implying that the format change did not affect binding to Fc receptors and C1q. The binding affinity of Fc receptors and C1q to the anti-CD20/CD20 BsAb was found to be in a similar range, as expected (**Figure 9B**).

## Discussion

Through this work, we have demonstrated that highly conserved interacting pairs of residues in the constant regions, C_H_1-C_L_ and C_H_3-C_H_3 (**Supplementary Figure 3**), can be modified to aid hetero-tetrameric IgG1-like BsAb assembly starting from the heavy and light chains of two monospecific antibodies. The mutations described herein help generate BsAbs at appreciable purity levels between 75-86%. While the platform in the current form is not ready to be deployed at a production scale, the mutations described herein can be combined with other *de novo* or published mutations to achieve purities >95%. Despite this limitation, there are several important elements this study offers that makes it unique. First, we are yet to come across a study that has tackled and developed solutions for the two problems: heavy chain heterodimerization and specific light chain association. Studies we have come across (to the best of our knowledge) have focused on one of these and employ known solutions (prior art) for the other (20, 46–49). Second, despite being a crowded space with multiple solutions offered by various groups (**Supplementary Tables 1**, **2**), the positions/mutations identified by this study are mostly unique, with only minimal overlap (e.g., K409R) (**Supplementary Tables 1**, **2**), indicating the vastness of the solution space and potential room to innovate in this field.

The antibody pairs (pertuzumab × DL11) selected for screening the Fab designs presented a challenging test case since their heavy and light chains had intrinsic potential for mispairing. In cases where the antibody combinations inherently favor cognate chain pairing over mispairing, we expect the assembly process to be more efficient due to the favorable chain association kinetics. It is noteworthy that the design of mutations did not involve comprehensive library-based screening (50, 51).

There are many instances where Fc-based immune effector functions would lead to undesirable toxicity (52). Such instances include BsAbs targeting a tumor cell and an immune cell, blockade of immune checkpoints on T lymphocytes, antibody-drug conjugate, targeting soluble ligands or inflammatory diseases. In these contexts, the IgG4 isotype is commonly employed as the *de facto* solution, as it shows weaker binding to C1q and Fcγ receptors (particularly to the activating FcγRIIIA), and hence, lower potential to mediate CDC and ADCC. We note that the mutations introduced in the constant regions are conserved in IgG4, suggesting portability of these mutations (**Supplementary Figure 4**).

Since the starting points for our computational modeling exercise employed kappa chains (pertuzumab and DL11), the solution space we derived is probably biased towards kappa antibodies and may not work in the same way on lambda antibodies. Indeed, the positions modified in C_L_′ and C_L_′′ are not conserved in lambda antibodies (123 is Gln in kappa but Glu in lambda, 136 is Asn in kappa but Ser in lambda, 177 is Thr in kappa but Tyr in lambda) hence they may not be transferable to lambda antibodies. Generating a solution space for lambda chains may require screening additional mutations which is beyond the scope of the current study.

Previous studies on rational design of BsAbs have mutated residues in the variable domains (53–55), possibly because the initial screening for interfacial mutations were made using antibodies lacking the variable domains (Fv), hence the specificity afforded by these mutations were insufficient to promote preferential pairing of whole heavy and light chains. By contrast, our screening strategy involved whole IgGs and therefore potentially resulted in selecting stronger C_H_1-C_L_ mutations, enabling 78 to 85% correct assembly. It is possible that the C_H_1-C_L_ mutations may not be as effective in driving the assembly if the heavy-light chain assembly was dominantly mediated by V_H_ and V_L_. In such cases, one would need to make changes to the variable regions, as documented before (46), to achieve the desired specificity. Our design approach relied on a combination of principles (charge, steric complementarity and hydrophobic interactions) to maximize the energy differences between target and off-target interactions. The effects of the hetero-tetrameric assembly on the antibody pharmacokinetics should be evaluated in animal models; however, initial biochemical assessment of our BsAbs in the established FcRn binding model indicates that the hetero-tetrameric assembly is unlikely to impact the antibody half-life (**Figure 9**).

Results from SDS-PAGE, SEC, and CD methods indicate that both anti-EGFR/HER2 and anti-CD20/CD20 BsAbs were formed correctly without deviating from the expected size and geometry. The correct heterodimer formation of BsAbs was also observed from mass spectrometry analysis The percent purity of these BsAbs with single step purification ranges from 78% to 85% as assessed by intact mass spectrometry. Collectively, these data indicate that our modifications at the C_H_1/C_L_ and C_H_3 interfaces provides the intended effect on chain assembly.

Protein thermal stability is one of the critical criteria for the selection of antibody drug candidates. The observed T_m_ values of the engineered anti-EGFR/HER2 and anti-CD20/CD20 BsAbs are similar to their parental mAbs, as well as in the same range as other IgGs, demonstrating that they are thermally stable and have less likelihood to aggregate (56–58). Moreover, the T_m_ values of both BsAbs from different lots of production were found to be reproducible (data not shown).

The choice of using a BsAb format will be determined primarily by whether the format translates to an improvement in efficacy in preclinical studies or clinical trials over mono or combination therapy (59). The clinical success of the few approved IgG-like BsAbs (60–64) along with innovation in low-cost manufacturing of biologics will continue to drive the demand for BsAbs. From a regulatory standpoint, the path for the approval of an IgG-like BsAb follows a trajectory similar to that of a monospecific antibody, making it both cost and time effective. In summary, our design principles lay the groundwork for developing an optimized platform for rapid generation of novel IgG-like BsAbs for diverse therapeutic areas.

## Data availability statement

The original contributions presented in the study are included in the article/**Supplementary Material**. Further inquiries can be directed to the corresponding authors.

## Author contributions

YI, KT, VS, MF and RS designed research. YI, KT, AK, AH, EF, and TR performed research; YI, KT, VS, AK, AH, PN-N, PT-O, and MF analyzed data, and YI, KT, VS, MR, JS, MF, and RS wrote the paper. All authors contributed to the article and approved the submitted version.

## Supplementary material

Supplementary Figure 1 Byproducts of co-expressing 2 heavy, 2 light chains of IgG.

Supplementary Figure 2 IgGs that were recombinantly expressed and quantified (by IgG ELISA) during the screening process to identify C_H_1 & C_L_ interface mutations. C_H_1’-C_L_’ and C_H_1”-C_L_” represent the cognate chain pairings (top and bottom species) whereas C_H_1’-C_L_” and C_H_1”-C_L_’ represent the non-cognate chain pairings (left and right species). Promising mutations favor the expression of matched or cognate IgG species and antagonize the formation of mispaired or non-cognate IgG species.

Supplementary Figure 3 Schematic diagram of the two BsAbs. **(A)** The anti-EGFR/HER2 BsAb contains variable regions specific to EGFR and HER2. Anti-EGFR variable regions were designed based on DL11 mAb, the anti-HER2 variable regions were designed based on pertuzumab mAb. **(B)** The anti-CD20/CD20 BsAb contains variable regions specific to CD20. The anti-CD20 variable regions were designed based on rituximab, which is a type I anti-CD20 monoclonal antibody and obinutuzumab, which is a type II anti-CD20 monoclonal antibody. The constant regions are based on human IgG1κ isotype.

Supplementary Figure 4 Conservation of heavy chain constant region mutations in IgG4 isotype. Residues modified for bispecific assembly are highlighted in red. The constant region of the light chain is conserved between IgG1 and IgG4. All the positions modified are conserved between the isotypes except 409, which is already a Arg in IgG4.

Supplementary Table 1 Fc mutations involved in bispecific platforms.

Supplementary Table 2 Fab mutations involved in bispecific platforms.

Supplementary Table 3 Interacting pairs of residues across the C_H_1-C_L_ interface.

Supplementary Table 4 SIN scores, BSA and distance from pseudo two-fold axis of C_H_1-C_L_ interface residues.

Supplementary Table 5 Rational structure-based designs of constant regions. Expression levels of matched and mismatched antibodies relative to their unmodified versions were computed in percentage form. ‘-’: expression not quantified due to missing heavy or light chain. ND: not determined. Columns 3 & 6 represent % expression levels of correctly paired species whereas columns 8 & 9 represent % expression levels of mispaired species (also refer **Figure S2**).

Supplementary Table 6 Rational structure-based designs of both the C_H_1_-_C_L_ mutations – relative expression of cognate heavy-light paired and mispaired IgGs.
