# Supplementary material for: Generation of bispecific antibodies by structure-guided redesign of IgG constant regions
Source: Front Immunol. 2023 Jan 10;13:1063002. doi: 10.3389/fimmu.2022.1063002 (PMC9871890; doi:10.3389/fimmu.2022.1063002)
Supplement: Supplementary Figure 1 — Byproducts of co-expressing 2 heavy, 2 light chains of IgG. [file DataSheet_1.pdf]

Supplementary Material

Generation of Bispecific Antibodies by Structure-Guided Redesign of IgG Constant Regions

Yordkhwan W. Iwasaki[1,†], Kannan Tharakaraman[2,†], Vidya Subramanian[2,†], Amnart Khongmanee[3], Andrew Hatas[2], Eduardo Fleischer[2], Troy T. Rurak[2], Patchara Ngok-ngam[3], Phanthakarn Tit-oon[3], Mathuros Ruchirawat[3,4], Jutamaad Satayavivad[1,4,5], Mayuree Fuangthong[3,4,6,*], and Ram Sasisekharan[2,7,*]

[1]Program in Environmental Toxicology, Chulabhorn Graduate Institute, Bangkok, Thailand

[2]Koch Institute for Integrative Cancer Research, Massachusetts Institute of Technology, Cambridge, MA, USA

[3]Translational Research Unit, Chulabhorn Research Institute, Bangkok, Thailand

[4]Center of Excellence on Environmental Health and Toxicology (EHT), OPS, MHESI, Thailand

[5]Laboratory of Pharmacology, Chulabhorn Research Institute, Bangkok, Thailand

[6]Program in Applied Biological Sciences, Chulabhorn Graduate Institute, Bangkok, Thailand

[7]Department of Biological Engineering, Massachusetts Institute of Technology, Cambridge, MA, USA

[†]These authors have contributed equally to this work and share first authorship

*** Correspondence:**
Mayuree Fuangthong, Ram Sasisekharan

mayuree@cri.or.th, rams@mit.edu

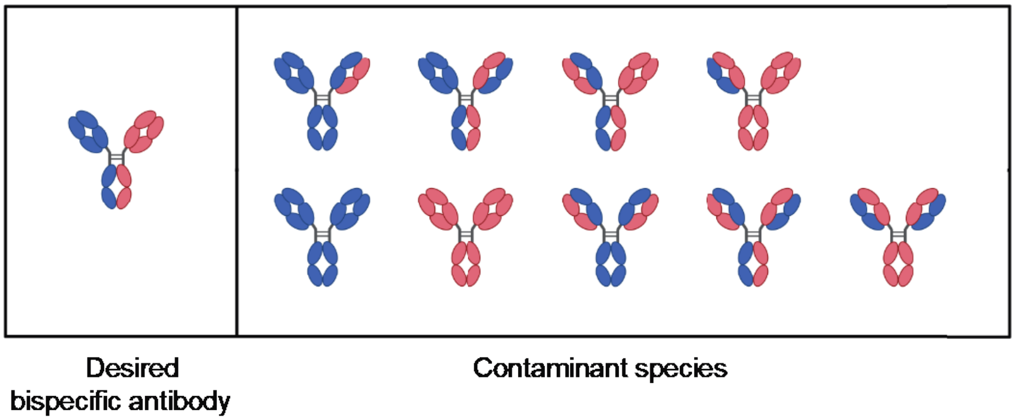

Figure S1. Byproducts of co-expressing 2 heavy, 2 light chains of IgG.

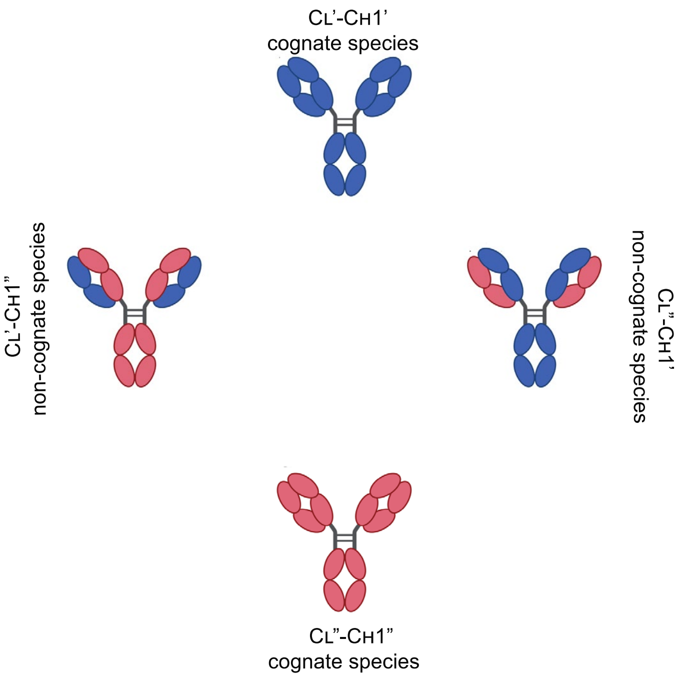

Figure S2. IgGs that were recombinantly expressed and quantified (by IgG ELISA) during the screening process to identify C_H1 & C_L interface mutations. C_H1'-C_L' and C_H1''-C_L'' represent the cognate chain pairings (top and bottom species) whereas C_H1'-C_L'' and C_H1''-C_L' represent the non-cognate chain pairings (left and right species). Promising mutations favor the expression of matched or cognate IgG species and antagonize the formation of mispaired or non-cognate IgG species.

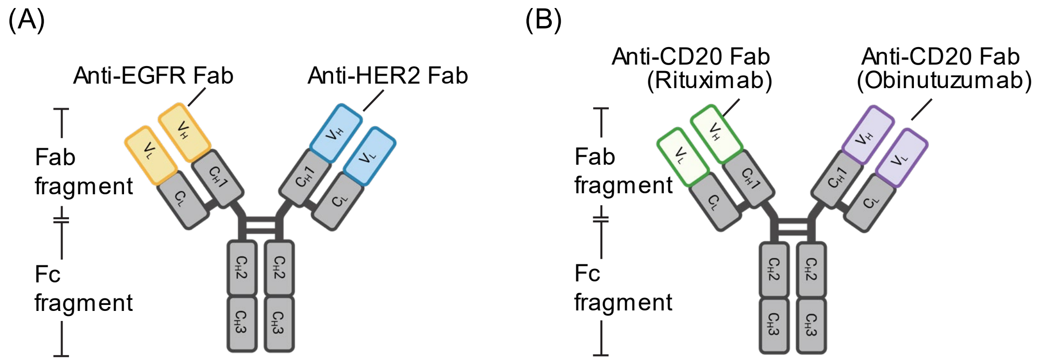

Figure S3. Schematic diagram of the two BsAbs. (A) The anti-EGFR/HER2 BsAb contains variable regions specific to EGFR and HER2. Anti-EGFR variable regions were designed based on DL11 mAb, the anti-HER2 variable regions were designed based on pertuzumab mAb. (B) The anti-CD20/CD20 BsAb contains variable regions specific to CD20. The anti-CD20 variable regions were designed based on rituximab, which is a type I anti-CD20 monoclonal antibody and obinutuzumab, which is a type II anti-CD20 monoclonal antibody. The constant regions are based on human IgG1κ isotype.

```
                       133                150-152                   173
H-GAMMA-4   ASTKGPSVFPLAPCSRSTSESTAALGCLVKDYFPEPVTVSWNSGALTSGVHTFPAVLQSS
H-GAMMA-1   ASTKGPSVFPLAPSSKSTSGGTAALGCLVKDYFPEPVTVSWNSGALTSGVHTFPAVLQSS
                 188
H-GAMMA-4   GLYSLSSVVTVPSSSLGTKTYTCNVDHKPSNTKVDKRVESKY-GPP--CPSCPAPEFLGG
H-GAMMA-1   GLYSLSSVVTVPSSSLGTQTYICNVNHKPSNTKVDKKVEPKSCDKTHTCPPCPAPELLGG

H-GAMMA-4   PSVFLFPPKPKDTLMISRTPEVTCVVVDVSQEDPEVQFNWYVDGVEVHNAKTKPREEQFN
H-GAMMA-1   PSVFLFPPKPKDTLMISRTPEVTCVVVDVSHEDPEVKFNWYVDGVEVHNAKTKPREEQYN
                                                                         357
H-GAMMA-4   STYRVVSVLTVLHQDWLNGKEYKCKVSNKGLPSSIEKTISKAKGQPREPQVYTLPPSQEE
H-GAMMA-1   STYRVVSVLTVLHQDWLNGKEYKCKVSNKALPAPIEKTISKAKGQPREPQVYTLPPSRDE
                         370                                   409
H-GAMMA-4   MTKNQVSLTCLVKGFYPSDIAVEWESNGQPENNYKTTPPVLDSDGSFFLYSRLTVDKSRW
H-GAMMA-1   LTKNQVSLTCLVKGFYPSDIAVEWESNGQPENNYKTTPPVLDSDGSFFLYSKLTVDKSRW

H-GAMMA-4   QEGNVFSCSVMHEALHNHYTQKSLSLSLGK
H-GAMMA-1   QQGNVFSCSVMHEALHNHYTQKSLSLSPGK
                               123         136
L-KAPPA-IGG4 RTVAAPSVFIFPPSDEQLKSGTASVVCLLNNFYPREAKVQWKVDNALQSGNSQESVTEQD
L-KAPPA-IGG1 RTVAAPSVFIFPPSDEQLKSGTASVVCLLNNFYPREAKVQWKVDNALQSGNSQESVTEQD

                        177
L-KAPPA-IGG4 SKDSTYSLSSTLTLSKADYEKHKVYACEVTHQGLSSPVTKSFNRGEC
L-KAPPA-IGG1 SKDSTYSLSSTLTLSKADYEKHKVYACEVTHQGLSSPVTKSFNRGEC
```

Figure S4. Conservation of heavy chain constant region mutations in IgG4 isotype. Residues modified for bispecific assembly are highlighted in red. The constant region of the light chain is conserved between IgG1 and IgG4. All the positions modified are conserved between the isotypes except 409, which is already a Arg in IgG4.

Table S1. Fc mutations involved in bispecific platforms.

HC1	HC2	Type	Source
Y349C, T366S, L368A, Y407V	S354C, T366W	Knobs into Holes + S-S stabilization SAV-W	Genentech (Merchant AM et al., Nat Biotechnol. 1998; PMID: 9661204)
E356K, E357K, D399K	K370E, K409D, K439E	Ionic, electrostatic	Chugai (WO2006106905)
K392D, K409D	E356K, D399K	Ionic, electrostatic DD-KK	Amgen (Gunasekaran K et al., J Biol Chem. 2010; PMID: 20400508)
S364H, F405A	Y349T, T394F	Mixed HA-TF	Xencor, Inc. (Moore GL et al., MAbs 2011; PMID: 22123055)
Fusion of leucine zipper A to C-terminus of CH3	Fusion of leucine zipper A to C-terminus of CH3	LUZ-Y	Max-Planck-Institute (Wranik BJ et al., J Biol Chem. 2012; PMID: 23118228)
IgG CH3	IgA CH3	SEEDbody	EMD Serono (Davis JH et al., Protein Eng Des Sel. 2010; PMID: 20299542)
F405L	K409R	Fab-arm exchange or DuoBody	Genmab (Labrijn AF et al., PNAS 2013; PMID: 23479652)
E345R, Q347R, T366V, K409V	K360D, D399M, Y407A	Hydrophobic/steric complementarity + electrostatic complementarity	Leaver-Fay A et al., Structure. 2016; PMID: 26996964
Y349S, K370Y, T366M, K409V	E356G, E357D, S364Q, Y407A	Negative-state repertoire	Leaver-Fay A et al., Structure. 2016; PMID: 26996964
T350V/L351Y/F405A/Y407V	T350V/T366L/K392L/T394W	Hydrophobic/steric complementarity	Zymeworks (Kreudenstein TSV et al., MAbs 2013; PMID: 23924797)
K370E	E357K, K409R	Inter-residue network	This study

Table S2. Fab mutations involved in bispecific platforms.

Heavy and light chain mutations	Format	Remarks	Type	Source
Q39, Q105 and S183 in heavy chain and Q38, A43 and S176 in light chain	4-chain Ig-like	Mutations in V_H/V_L	Electrostatic steering	Amgen (US9822173B2)
V37F and L45W in heavy chain and Y87A and F98M in light chain	V_H-V_L heterodimer	Mutations in V_H/V_L	Hydrophobic/Steric	Genentech (Zhu Z et al., Protein Sci. 1997; PMID: 9098887)
Q39E/K in heavy chain and Q38K/E in light chain	Single chain diabody	Mutations in V_H/V_L	Electrostatic	Chugai (WO2006106905)
V37, Q39, W103, F100, A139, L143, D144, K145, D146, F174, P175, Q179, S188 and V190 in heavy chain<br>And Q38, P44, T85, F98, F116, F118, Q124, V133, L135, Q160, S176, T178 and T180 in light chain	4-chain Ig-like	Mutations in V_H/V_L	Unkown	Zymeworks (WO2015181805)
Q39K/Y, R62E, H172A, F174G, V190 in heavy chain and D1R, Q38D/R, L135Y, S176W in light chain	4-chain Ig-like	Mutations in V_H/V_L	Steric/Charge	Eli Lilly (Lewis SM et. al., Nat Biotechnol. 2014; PMID: 24463572)
39K and TCRCa in heavy chain and 38D and TCR Cb in light chain	4-chain Ig-like	Mutations in V_H/V_L	IgG/TCR chimeras for specific pairing	Eli Lilly (Wu X et al., MAbs 2015; PMID: 25611120)
T192E, L143Q, S188V in heavy chain and N137K, S114A, V133T and S176V in light chain	Tetravalent	No mutations in V_H/V_L	Charged residues and hydrophobicity-polarity-swap	Golay J et al. J Immunol. 2016; PMID: 26921308
No mutations	4-chain Ig-like	NA	Doman crossover (CrossMab)	Schaefer W et al., PNAS 2011; PMID: 21690412
A20L, K26D in heavy chain and F7S/A/V, T18R in light chain	4-chain Ig-lie	No mutations in V_H/V_L	Predictions were made using FoldX	Bönisch M et al., Protein Eng Des Sel. 2017; PMID: 28981885
F126C in heavy chain and S121C in light chain	DuetMab	No mutations in V_H/V_L	Engineered disulfide bond	Mazor Y et al., MAbs 2015; PMID: 25621507
L133V, L150A, K152D, H173D, S188W in heavy chain and Q123, N136, T177 in light chain	4-chain Ig-like	Kappa-specific	Steric/Charge/Hydrophobic	This study

Table S3. Interacting pairs of residues across the C_H1-C_L interface.

C_H1 residue	Interaction	C_L residue
LEU-133	Hydrophobic interaction	PHE-117
LEU-133	Hydrophobic interaction	VAL-132
ALA-134	Hydrophobic interaction	PHE-117
PRO-135	Hydrophobic interaction	PHE-117
LYS-138	Hydrogen bond	GLU-212
LYS-138	Ionic bond	GLU-212
LYS-138	Cation-pi interaction	PHE-208
ALA-146	Hydrophobic interaction	PHE-117
ALA-146	Hydrophobic interaction	PHE-115
ALA-146	Hydrophobic interaction	LEU-134
LEU-147	Hydrophobic interaction	PHE-117
LEU-150	Hydrophobic interaction	VAL-132
HIS-173	Hydrogen bond	ASN-137
HIS-173	Hydrogen bond	SER-173
HIS-173	Hydrogen bond	ASN-136
HIS-173	Ionic bond	ASP-166
PHE-175	Hydrophobic interaction	VAL-162
PHE-175	Hydrophobic interaction	LEU-174
PHE-175	Hydrophobic interaction	LEU-134
PRO-176	Hydrophobic interaction	VAL-162
PRO-176	Hydrogen bond	SER-161
LEU-179	Hydrogen bond	GLN-159
VAL-190	Hydrophobic interaction	LEU-134
LYS-218	Ionic bond	GLU-122
LYS-223	Hydrogen bond	CYS-213
LYS-223	Ionic bond	ASP-121
CYS-225	Disulfide bridge	CYS-213

Table S4. SIN scores, BSA and distance from pseudo two-fold axis of C_H1-C_L interface residues.

Residue	SIN score	Buried Surface Area	Distance from two-fold axis
H:LEU-133	0.275	0.939	5.774
H:ALA-134	0.053	0.69	9.211
H:PRO-135	0.188	0	10.247
H:LYS-138	0.256	0.723	8.699
H:ALA-146	0.071	1	8.025
H:LEU-147	0.411	0.915	7.705
H:LEU-150	0.345	0.901	0.822
H:HIS-173	0.275	0.773	9.827
H:PHE-175	0.443	0.982	8.609
H:PRO-176	0.125	0.379	11.092
H:LEU-179	0.221	0.101	13.29
H:VAL-190	0.243	0.945	6.203
H:LYS-218	0.231	0.258	9.364
H:LYS-223	0.178	0.6	15.041
H:CYS-225	0.137	0.57	16.429
L:PHE-115	0.447	0.95	2.808
L:PHE-117	0.432	0.996	6.073
L:ASP-121	0.101	0.134	13.432
L:GLU-122	0.26	0.427	11.584
L:VAL-132	0.27	0.962	4.415
L:LEU-134	0.323	0.994	1.579
L:ASN-136	0.257	0.803	6.504
L:ASN-137	0.158	0.266	10.078
L:GLN-159	0.133	0.49	11.465
L:SER-161	0.135	0.846	9.926
L:VAL-162	0.262	0.368	11.35
L:ASP-166	0.175	0.209	17.312
L:SER-173	0.207	0.991	9.019
L:LEU-174	0.303	0.316	7.507
L:PHE-208	0.715	0.5	12.454
L:GLU-212	0.162	0.15	18.275
L:CYS-213	0.073	0.664	16.696

Table S5. Rational structure-based designs of constant regions. Expression levels of matched and mismatched antibodies relative to their unmodified versions were computed in percentage form. '-': expression not quantified due to missing heavy or light chain. ND: not determined. Columns 3 & 6 represent % expression levels of correctly paired species whereas columns 8 & 9 represent % expression levels of mispaired species (also refer Fig. S2).

**Cluster**	**Pertuzumab CORRECT PAIRING**			**DL11 CORRECT PAIRING**			**MISPAIRING**	
	**Pertuzumab**			**DL11**				
	**C_L'**	**% Expression (C_L'-C_H1')**	**C_H1'**	**C_L''**	**% Expression (C_L''-C_H1'')**	**C_H1''**	**C_L''-C_H1'**	**C_L'-C_H1''**
1	WT	100%	WT	117V, 132D, 134D	0%	133Q, 175K, 188N, 190N	0%	14%
2	WT	100%	WT	117S, 132D, 134D	1%	133Q, 175K, 188N, 190N	0%	14%
3	WT	100%	WT	132D, 134D	0%	133Q, 175K, 188N, 190N	7%	14%
4	WT	100%	WT	134D	0%	133Q, 175K, 188N, 190N	63%	14%
5	WT	100%	WT	132D	0%	133Q, 175K, 188N, 190N	39%	14%
6	WT	100%	WT	117S, 132D, 134D	0%	133Q, 146Q, 175R, 188N, 190Q	0%	6%
7	WT	100%	WT	117V, 132D, 134D	0%	133Q, 146Q, 175R, 188N, 190Q	0%	6%
8	WT	100%	WT	132D, 134D	0%	133Q, 146Q, 175R, 188N, 190Q	7%	6%
9	WT	100%	WT	134D	0%	133Q, 146Q, 175R, 188N, 190Q	63%	6%
10	WT	100%	WT	132D	0%	133Q, 146Q, 175R, 188N, 190Q	39%	6%
11	WT	100%	WT	132D, 134D	3%	175Y	159%	ND
12	WT	100%	WT	134D	47%	175Y	ND	ND
13	WT	100%	WT	132D	21%	175Y	ND	ND
14	WT	100%	WT	132D, 134D	0%	175Y, 190A	110%	ND
15	WT	100%	WT	134D	11%	175Y, 190A	ND	ND
16	WT	100%	WT	132D	4%	175Y, 190A	ND	ND
17	177R	64%	150D	134D	0%	150Y, 190N	12%	**0%**
18	177R	18%	150D, 190K	134D	12%	150Y, 190R	0%	**9%**
19	177R	63%	150D	134D	55%	WT	10%	64%
20	177R	18%	150D, 190K	134D	58%	175Y	0%	84%
21	177R	-	Not included	134D	0%	150Y, 190N	-	0%
22	177R	-	Not included	134D	12%	150Y, 190R	-	9%
23	159K	109%	180D	WT	108%	180K	104%	62%

Cluster	Pertuzumab CORRECT PAIRING			DL11 CORRECT PAIRING			MISPAIRING	
	Pertuzumab			DL11				
	C_L'	% Expression (C_L'-C_H1')	C_H1'	C_L''	% Expression (C_L''-C_H1'')	C_H1''	C_L''-C_H1'	C_L'-C_H1''
24	123E,159K, 177K	127%	180D	123K, 159E,177D	**2%**	180K	**2%**	132%
25	Not included	-	180D	123K, 159E	29%	180K	**95%**	-
26	Not included	-	180D	123K	20%	180K	**98%**	-
27	Not included	-	180D	159D	120%	180K	**104%**	-
28	177K	ND	180E	166H, 177E	ND	173D, 180K	110%	73%
29	177K	ND	173K, 180E	166K, 177E	ND	173D, 180K	99%	73%
30	WT	100%	WT	122K, 212K	102%	138E, 218E	74%	106%
31	123D,136D	104%	152H	123H, 136H	45%	152D, 173D	76%	37%
32	123D,136D	73%	WT	123K, 136K	84%	152D, 173D	***47%***	37%
33	123D,136D	78%	WT	123K, 136K	68%	152D, 173D	**74%**	31%
34	159K		180D	159D	120%	180K	104%	62%
35	132F	90%	133V, 150A	177A	85%	188F	41%	91%
36	132W	96%	133V, 150A	177A	85%	188W	41%	85%
37	WT	100%	WT	134W	11%	146G, 175A, 190G	60%	90%
38	WT	100%	WT	123W	36%	131A, 150A	40%	140%
39	WT	100%	WT	123W	66%	131A, 150V	40%	119%
40	123D, 136D	87%	133V, 150A	123K, 136K, 177A	63%	152D, 173D, 188W	0%	48%
41	123D, 136D, 177R	62%	150D	123K, 136K	86%	152D, 173D	54%	43%
42	177R	52%	150D	134D	48%	WT	10%	48%
43	123D, 136D, 177R	45%	150D	123K, 134D, 136K	4%	152D, 173D	0%	40%
44	123D, 132W, 136D	43%	133V, 150A	123K, 136K, 177A	57%	152D, 173D, 188W	0%	23%

Table S6. Rational structure-based designs of both the C_H1-C_L mutations – relative expression of cognate heavy-light paired and mispaired IgGs.

Mutation Set	Lot Name	Heavy Chain	Light Chain	Expression (µg/mL)	%Correct C_H1/C_L	%Incorrect C_H1/C_L	Other Description
Pertuzumab/ DL11	AFM5	Pertuzumab WT	Pertuzumab WT	59.366	58.45%	41.55%	3-Chain Total H:L 1:1
			DL11 WT				
	AFL5	Pertuzumab (133V, 150A)	Pertuzumab (123D, 136D)	10.23	100.00%	0.00%	
			DL11 (123K, 136K, 177A)				
	AFM6		Pertuzumab WT	54.127	43.75%	56.25%	
		DL11 WT	D L11 WT				
	AFL6		Pertuzumab (123D, 136D)	50.151	55.05%	44.95%	
		DL11 (152D, 173D, 188W)	DL11 (123K, 136K, 177A)				
	AFM7	Pertuzumab WT	Pertuzumab WT	68.486	54.40%	45.60%	
		DL11 WT					
	AFL7	Pertuzumab (133V, 150A)	Pertuzumab (123D, 136D)	42.122	48.90%	51.20%	
		DL11 (152D, 173D, 188W)					
	AFM8	Pertuzumab WT		56.392	48.20%	51.80%	
		DL11 WT	DL11 WT				
	AFL8	Pertuzumab (133V, 150A)		6.514	100.00%	0.00%	
		DL11 (152D, 173D, 188W)	DL11 (123K, 136K, 177A)				

Table S7. Predicted number of weak and strong binders of MHC II alleles in the WT and modified constant regions.

	WT			Mutants		
	**Chain**	**Weak binders**	**Strong binders**	**Chain**	**Weak binders**	**Strong binders**
**mAb1**	Heavy	202	43	Heavy - L133V, L150A, E357K, K409R	199	43
	Light	74	32	Light - Q123D, N136D	73	32
**mAb2**	Heavy	202	43	Heavy - K152D, H173D, S188W, K370E	184	35
	Light	74	32	Light - Q123K, N136K, T177A	83	34
Total		552	150		539	144

Table S8. Amino acid sequencing results of the bispecific antibodies that were used for analytical and functional characterization. Mutations introduced to form a bispecific assembly are listed and marked in the sequence (in red bold font).

Bispecific	Chains	Mutations	Sequence
Anti-HER2 X HER1/HER3	Pertuzumab-Heavy Chain	L133V, L150A, E357K, K409R	EVQLVESGGGLVQPGGSLRLSCAASGFTFTDYTMDWVRQAPGKGLEWVADVNPNSGGSIYNQRFKGRFTLSVDRSKNTLYLQMNSLRAEDTAVYYCARNLGPSFYFDYWGQGTLVTVSSASTKGPSVFP**V**APSSKSTSGGTAALGC**A**VKDYFPEPVTVSWNSGALTSGVHTFPAVLQSSGLYSLSSVVTVPSSSLGTQTYICNVNHKPSNTKVDKKVEPKSCDKTHTCPPCPAPELLGGPSVFLFPPKPKDTLMISRTPEVTCVVVDVSHEDPEVKFNWYVDGVEVHNAKTKPREEQYNSTYRVVSVLTVLHQDWLNGKEYKCKVSNKALPAPIEKTISKAKGQPREPQVYTLPPSRD**K**LTKNQVSLTCLVKGFYPSDIAVEWESNGQPENNYKTTPPVLDSDGSFFLYS**R**LTVDKSRWQQGNVFSCSVMHEALHNHYTQKSLSLSPGK
	Pertuzumab-Light Chain	Q123D, N136D	DIQMTQSPSSLSASVGDRVTITCKASQDVSIGVAWYQQKPGKAPKLLIYSASYRYTGVPSRFSGSGSGTDFTLTISSLQPEDFATYYCQQYYIYPYTFGQGTKVEIKGSVAAPSVFIFPPSDE**D**LKSGTASVVCLL**D**NFYPREAKVQWKVDNALQSGNSQESVTEQDSKDSTYSLSSTLTLSKADYEKHKVYACEVTHQGLSSPVTKSFNRGEC
	DL11-Heavy Chain	K152D, H173D, S188W, K370E	EVQLVESGGGLVQPGGSLRLSCAASGFTLSGDWIHWVRQAPGKGLEWLGEISAAGGYTDYADSVKGRFTISADTSKNTAYLQMNSLRAEDTAVYYCARESRVSFEAAMDYWGQGTLVTVSSASTKGPSVFPLAPSSKSTSGGTAALGCLV**D**DYFPEPVTVSWNSGALTSGV**D**TFPAVLQSSGLYSL**W**SVVTVPSSSLGTQTYICNVNHKPSNTKVDKKVEPKSCDKTHTCPPCPAPELLGGPSVFLFPPKPKDTLMISRTPEVTCVVVDVSHEDPEVKFNWYVDGVEVHNAKTKPREEQYNSTYRVVSVLTVLHQDWLNGKEYKCKVSNKALPAPIEKTISKAKGQPREPQVYTLPPSRDELTKNQVSLTCLV**E**GFYPSDIAVEWESNGQPENNYKTTPPVLDSDGSFFLYSKLTVDKSRWQQGNVFSCSVMHEALHNHYTQKSLSLSPGK

Bispecific	Chains	Mutations	Sequence
	DL11-Light Chain	Q123K, N136K, T177A	DIQMTQSPSSLSASVGDRVTITCRASQDLATD<br>VAWYQQKPGKAPKLLIYSASFLYSGVPSRFSG<br>SGSGTDFTLTISSLQPEDFATYYCQQSEPEPY<br>TFGQGTKVEIKGSVAAPSVFIFPPSDE**K**LKSG<br>TASVVCLL**K**NFYPREAKVQWKVDNALQSGNSQ<br>ESVTEQDSKDSTYSLSS**A**LTLSKADYEKHKVY<br>ACEVTHQGLSSPVTKSFNRGEC
Anti-CD20 X CD20	Rituximab-Heavy Chain	L133V, L150A, E357K, K409R	QVQLQQPGAELVKPGASVKMSCKASGYTFTSY<br>NMHWVKQTPGRGLEWIGAIYPGNGDTSYNQKF<br>KGKATLTADKSSSTAYMQLSSLTSEDSAVYYC<br>ARSTYYGGDWYFNVWGAGTTVTVSAASTKGPS<br>VFP**V**APSSKSTSGGTAALGC**A**VKDYFPEPVTV<br>SWNSGALTSGVHTFPAVLQSSGLYSLSSVVTV<br>PSSSLGTQTYICNVNHKPSNTKVDKKVEPKSC<br>DKTHTCPPCPAPELLGGPSVFLFPPKPKDTLM<br>ISRTPEVTCVVVDVSHEDPEVKFNWYVDGVEV<br>HNAKTKPREEQYNSTYRVVSVLTVLHQDWLNG<br>KEYKCKVSNKALPAPIEKTISKAKGQPREPQV<br>YTLPPSRD**K**LTKNQVSLTCLVKGFYPSDIAVE<br>WESNGQPENNYKTTPPVLDSDGSFFLYS**R**LTV<br>DKSRWQQGNVFSCSVMHEALHNHYTQKSLSLS<br>PGK
	Rituximab-Light Chain	Q123D, N136D	QIVLSQSPAILSASPGEKVTMTCRASSSVSYI<br>HWFQQKPGSSPKPWIYATSNLASGVPVRFSGS<br>GSGTSYSLTISRVEAEDAATYYCQQWTSNPPT<br>FGGGTKLEIKGSVAAPSVFIFPPSDE**D**LKSGT<br>ASVVCLL**D**NFYPREAKVQWKVDNALQSGNSQE<br>SVTEQDSKDSTYSLSSTLTLSKADYEKHKVYA<br>CEVTHQGLSSPVTKSFNRGEC
	Obinutuzumab-Heavy Chain	K152D, H173D, S188W, K370E	QVQLVQSGAEVKKPGSSVKVSCKASGYAFSYS<br>WINWVRQAPGQGLEWMGRIFPGDGDTDYNGKF<br>KGRVTITADKSTSTAYMELSSLRSEDTAVYYC<br>ARNVFDGYWLVYWGQGTLVTVSSASTKGPSVF<br>PLAPSSKSTSGGTAALGCLV**D**DYFPEPVTVSW<br>NSGALTSGV**D**TFPAVLQSSGLYSL**W**SVVTVPS<br>SSLGTQTYICNVNHKPSNTKVDKKVEPKSCDK<br>THTCPPCPAPELLGGPSVFLFPPKPKDTLMIS<br>RTPEVTCVVVDVSHEDPEVKFNWYVDGVEVHN<br>AKTKPREEQYNSTYRVVSVLTVLHQDWLNGKE<br>YKCKVSNKALPAPIEKTISKAKGQPREPQVYT<br>LPPSRDELTKNQVSLTCLV**E**GFYPSDIAVEWE<br>SNGQPENNYKTTPPVLDSDGSFFLYSKLTVDK

Bispecific	Chains	Mutations	Sequence
			SRWQQGNVFSCSVMHEALHNHYTQKSLSLSPGK
	Obinutuzumab-Light Chain	Q123K, N136K, T177A	DIVMTQTPLSLPVTPGEPASISCRSSKSLLHSNGITYLYWYLQKPGQSPQLLIYQMSNLVSGVPDRFSGSGSGTDFTLKISRVEAEDVGVYYCAQNLELPYTFGGGTKVEIKGSVAAPSVFIFPPSDE**K**LKSGTASVVCLL**K**NFYPREAKVQWKVDNALQSGNSQESVTEQDSKDSTYSLSS**A**LTLSKADYEKHKVYACEVTHQGLSSPVTKSFNRGEC

Table S9. Summary of all possible molecular weights and observable percentage for each BsAb. All theoretical masses are calculated using Agilent MassHunter Sequence Manager B.09.00 (Agilent Technologies). All theoretical masses shown are C-terminal lysine clipping variants.

Possible assemblies	anti-EGFR/HER2 BsAb			anti-CD20/CD20 BsAb		
	Theoretical mass (Da)	Observed mass (Da)	% Found	Theoretical mass (Da)	Observed mass (Da)	% Found
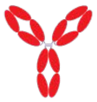	anti-EGFR monospecific 144370.66	144366.56	0.5	anti-CD20 monospecific (1) 143936.45	143935.06	0.7
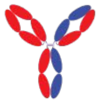	144433.98	ND	ND	144021.49	ND	ND
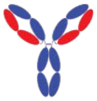	144497.24	ND	ND	144106.52	ND	ND
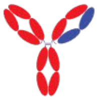	144535.81	ND	ND	145064.09	ND	ND
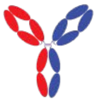	BsAb 144599.10	144597.05	84.8	BsAb 145149.12	145146.85	78.3
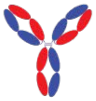	144599.10	ND	ND	145149.12	ND	ND
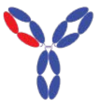	144662.39	ND	ND	145234.16	ND	ND
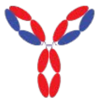	144700.96	144705.24	1.4	146191.72	ND	ND
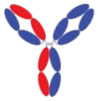	144764.25	144759.92	13.3	146276.76	ND	ND
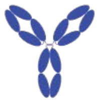	anti-HER2 monospecific 144827.54	144824.95	0.1	anti-CD20 monospecific (2) 146361.79	146359.23	21

ND = Not detected
